# Risk Factors for Delirium in the Palliative Care Population: A Systematic Review and Meta-Analysis

**DOI:** 10.3389/fpsyt.2021.772387

**Published:** 2021-10-21

**Authors:** Duan Guo, Taiping Lin, Chuanyao Deng, Yuxia Zheng, Langli Gao, Jirong Yue

**Affiliations:** ^1^Department of Geriatrics and National Clinical Research Center for Geriatrics, West China Hospital, Sichuan University, Chengdu, China; ^2^Department of Palliative Medicine, West China School of Public Health and West China Fourth Hospital, Sichuan University, Chengdu, China; ^3^West China School of Nursing, Sichuan University, Chengdu, China; ^4^Department of Geriatrics, West China Hospital, Sichuan University, Chengdu, China

**Keywords:** delirium, risk factors, palliative care, systematic review, meta-analysis

## Abstract

**Objective:** Delirium is common and highly distressing for the palliative care population. Until now, no study has systematically reviewed the risk factors of delirium in the palliative care population. Therefore, we performed a systematic review and meta-analysis to evaluate delirium risk factors among individuals receiving palliative care.

**Methods:** We systematically searched PubMed, Medline, Embase, and Cochrane database to identify relevant observational studies from database inception to June 2021. The methodological quality of the eligible studies was assessed by the Newcastle Ottawa Scale. We estimated the pooled adjusted odds ratio (aOR) for individual risk factors using the inverse variance method.

**Results:** Nine studies were included in the review (five prospective cohort studies, three retrospective case-control studies and one retrospective cross-section study). In pooled analyses, older age (aOR: 1.02, 95% CI: 1.01–1.04, *I*^2^ = 37%), male sex (aOR:1.80, 95% CI: 1.37–2.36, *I*^2^ = 7%), hypoxia (aOR: 0.87, 95% CI: 0.77–0.99, *I*^2^ = 0%), dehydration (aOR: 3.22, 95%CI: 1.75–5.94, *I*^2^ = 18%), cachexia (aOR:3.40, 95% CI: 1.69–6.85, *I*^2^ = 0%), opioid use (aOR: 2.49, 95%CI: 1.39–4.44, *I*^2^ = 0%), anticholinergic burden (aOR: 1.18, 95% CI: 1.07–1.30, *I*^2^ = 9%) and Eastern Cooperative Oncology Group Performance Status (aOR: 2.54, 95% CI: 1.56–4.14, *I*^2^ = 21%) were statistically significantly associated with delirium.

**Conclusion:** The risk factors identified in our review can help to highlight the palliative care population at high risk of delirium. Appropriate strategies should be implemented to prevent delirium and improve the quality of palliative care services.

## Introduction

Delirium is a complex neuropsychiatric syndrome characterized by acute onset and fluctuating disturbance in attention, awareness, and other cognitive abilities, including memory, orientation, language, and perception (1, 2). Delirium is commonly experienced during individuals receiving palliative care. The prevalence of delirium in palliative care settings has been reported at a range of 28–42% on admission, whereas it can rise to 88% before death (3, 4). Delirium is known to be associated with reduced quality of life, shortened survival expectancy, and increased health care costs (5, 6), which is highly distressing for the palliative care population, caregivers, as well as clinicians (7).

The etiology of delirium is multifactorial, and the pathophysiologic cause is not well-understood (8). It is important to recognize risk factors for delirium, which could promote the early identification, prevention, and treatment of delirium. Palliative care services are usually provided for patients with cancer or organ failure who are susceptible to delirium. However, despite the high occurrence of delirium in palliative care units, only very few studies investigate the risk factors in this vulnerable population, and the results from different studies are inconsistent (9, 10). Until now, the contributing factors of delirium in the palliative care population remain unclear, so it is necessary to perform a comprehensive evaluation of this area.

Until now, there has not been a systematic review to evaluate risk factors of delirium in the palliative care population. Therefore, the aims of our systematic review and meta-analysis were (a) to identify risk factors of delirium among individuals receiving palliative care; (b) to examine the methodology and quality of included studies.

## Methods

We did a systematic review and meta-analysis following the recommendations of Preferred Reporting Items for Systematic Reviews and Meta-analyses guidelines (PRISMA) (11).

### Search Strategy

We systematically searched PubMed, Medline, Embase, and Cochrane databases to identify relevant observational studies without language restrictions from database inception to June 2021. The words “delirium” and “palliative care” were used as key terms for searching. The detailed search strategy for MEDLINE was reported in the supplementary materials (Supplementary Table 1). We also hand-searched the reference lists of eligible studies and previous relevant reviews to identify additional studies.

### Study Selection

Studies were independently selected by two reviewers (DG and TL) using a two-stage process. First, the titles and abstracts of studies were screened, and then the full text of screened articles was assessed for eligibility. Any discrepancy regarding the screening and selection of studies or the following data extraction was resolved through consensus with a third independent reviewer (JY).

The selection criteria were as follows: (a) observational studies (cross-sectional, cohort, and case-control studies); (b) primary research evaluating potential risk factors including predisposing factors and precipitating factors for prevalent delirium or incident delirium; (c) studies with validated instruments or criteria to identify delirium; (d) studies conducted within any palliative care settings: inpatient palliative care units, stand-alone inpatient hospices, hospital palliative care teams, and community palliative care services (12); (e) studies with two comparator groups: a delirium group and a non-delirium group; (f) risk factors analyzed in multivariable models. Studies were excluded if: (a) they were case reports, letters, comments, editorials, reviews, and protocols; (b) they had incomplete or unavailable data.

### Data Extraction and Analysis

Data from all eligible studies were independently extracted by two reviewers (DG and YZ). Extracted information included the first author's name, publication year, country, number of patients, male proportion, age, study design, prevalence or incidence of delirium, criteria for delirium, and potential risk factors for delirium. A third reviewer (LG) reviewed the data extraction, and any disagreement was resolved by discussion. We contacted authors when the relevant information was not available in the publication. If the risk factors for delirium were assessed at admission and during admission simultaneously, the adjusted effect measures during admission were extracted.

We conducted the meta-analysis if two or more studies evaluated a risk factor using a multivariable approach and available data. We estimated the pooled adjusted odds ratios (aORs) using the inverse variance method in Review Manager (Version 5.4). We used the *I*^2^ test to assess the statistical heterogeneity. A random-effects model was chosen when statistical heterogeneity was present (*I*^2^ ≥ 50%). Otherwise, a fixed-effect model was applied. For heterogeneity, we performed subgroup analysis or sensitivity analysis to investigate possible causes of heterogeneity. If more than five studies were eligible for comparison, funnel plot and Egger's test were applied to assess publication bias (13).

### Quality Assessment

The quality of the included studies was evaluated independently by two reviewers (DG and TL) using the Newcastle–Ottawa Scale (NOS) (14), which assessed the quality of observational studies in three aspects (selection, comparability, and exposure/outcomes). The total score was classified as high quality (≥7 points), moderate quality (5–6 points), or low quality (≤4 points). Any disagreement about the quality of the studies was resolved by a third reviewer (JY) through discussion.

## Results

### Study Selection

**Figure 1:**
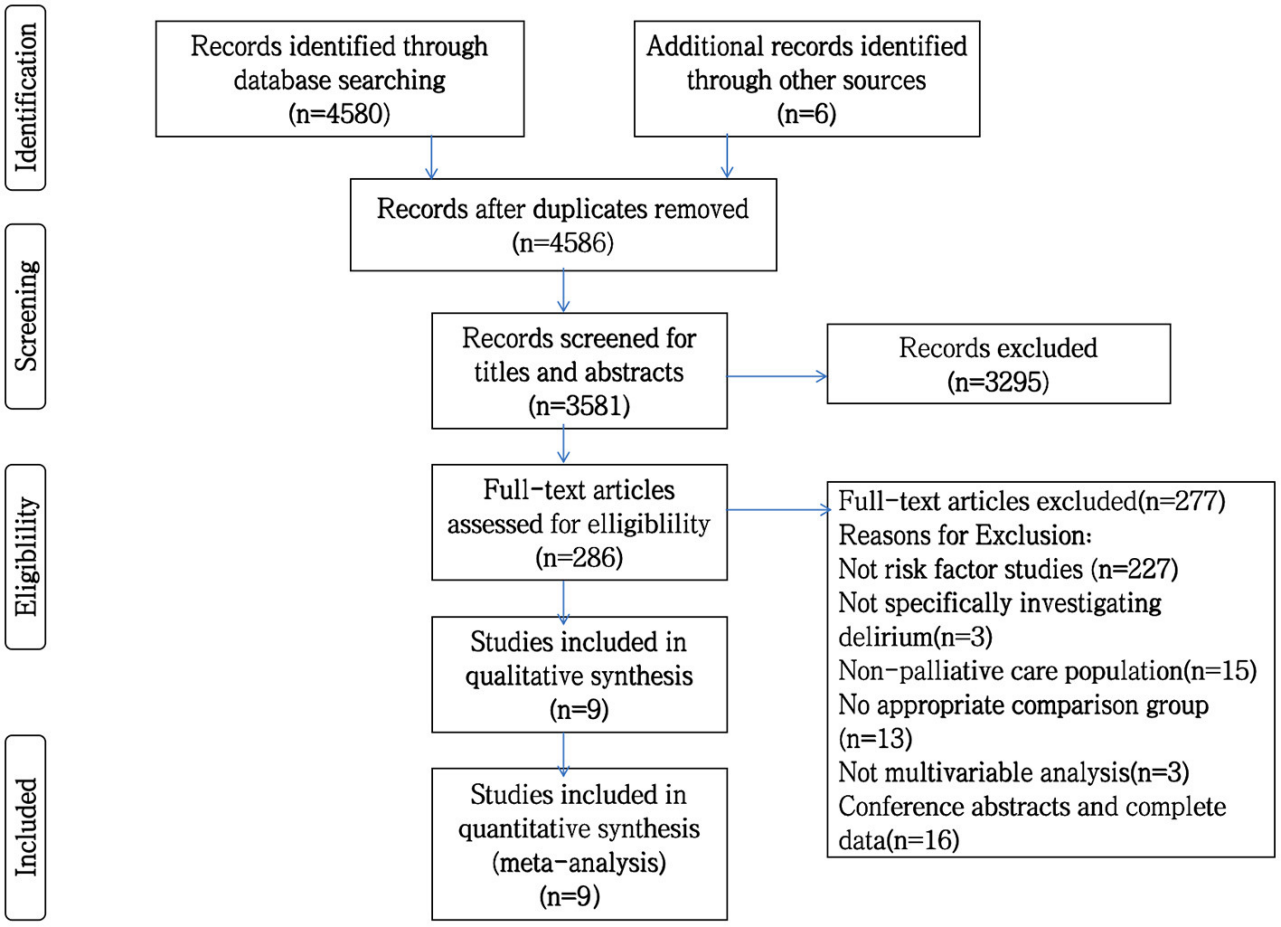
PRISMA flowchart of studies included in systematic review.

A total of 4,586 studies were identified through the initial electronic search and manual review of references. After removing duplicates, 3,581 studies remained. Of these, 3,295 studies were excluded by screening the title and abstract. The remaining 286 studies were assessed by full-text review, and nine were finally included in our synthesis. Figure 1 shows the flowchart and details of the study selection process.

### Study Characteristics

The characteristics of the nine included studies were presented in Table 1 (9, 10, 15–21). Among these studies, three were case-control studies (9, 19, 20), and five were cohort studies (10, 15–18). The studies involved 4,939 subjects (899 delirium cases and 4,040 non-delirious controls) from five countries. Four studies were conducted in Japan (9, 10, 15, 20), two studies in Italy (18, 21), and one each from United States (19), Switzerland (17), and Korea (16). Three multicenter prospective studies enrolled patients receiving palliative care services (10, 15, 18). A retrospective cross-sectional study was conducted in end-of-life patients in a hospice or cared for at home by palliative care physicians (21). Another study retrospective reviewed palliative care inpatients admitted to the Veterans Affairs Boston Healthcare System (19). The remaining four single-center studies were conducted in hospital-based palliative care units (9, 16, 17) or hospice-based palliative care units (20).

**Table 1:** Characteristics of the included studies examining risk factors for delirium in palliative population.

**References**	**Country**	**Study design**	**Total number**	**Age(mean ± SD)unless otherwise stated**	**No. of male**	**Delirium prevalence or incidence(%)**	**Criteria for delirium**	**Delirium subtype**	**NOS**
Hamano et al. (15)	Japan	Prospective cohort	2,829	72.4 ± 12.2	1,492	6.9	DSM-V, MDAS	Hyperactive type	6
Kang et al. (16)	Korea	Prospective cohort	102	Delirium group: 71.83 ± 9.82 Non-delirium group: 62.04 ± 14.35	52	23.52	CAM, DSM-IV	No mention	6
Seiler et al. (17)	Switzerland	Prospective cohort	410	Delirium group: 66.4 ± 14.1 Non-delirium group: 64.5 ± 12.4	244	55.9	DOS, DSM-V	No mention	6
Matsuo et al. (10)	Japan	Prospective cohort	207	Median:73	98	17	CAM	No mention	6
Mercadante et al. (18)	Italy	Prospective cohort	263	72.1 ± 13.7	146	41.8	MDAS	No mention	8
Matsuoka et al. (9)	Japan	Case Control	166	68.4 ± 11.6	97	35	DSM-IV	Hyperactive and mixed type	5
Zimmerman et al. (19)	United state	Case control	217	72.9 ± 12.8	210	31	A chart review instrument	No mention	7
Morita et al. (20)	Japan	Case Control	284	64 ± 14	153	20.4	MDAS, Agitation Distress Scale	Hyperactive type	6
Pasina et al. (21)	Italy	Cross-section	461	Median of delirium group:82.6 median of non-delirium group:78.1	226	26.9	4AT	No mention	5

*NOS, Newcastle-Ottawa Scale; DSM, Diagnostic and Statistical Manual of Mental Disorders; CAM, Confusion Assessment Method; MDAS, Memorial Delirium Assessment Scale; DOS, Delirium Observation Screening; 4AT, 4 “A”s Test*.

Two studies explored the risk factors for hyperactive delirium only (15, 20), while another study investigated risk factors for hyperactive and mixed-type delirium (9). The remaining six studies did not mention the subtype of delirium (10, 17–19, 21). Six studies reported the mean age of study participants from 64 to 72.9 years (9, 10, 15, 18–20). All studies reported gender: 2,718 (55.03%) males, and 2,221 (44.97%) were female. Tools used to identify delirium were the Diagnostic and Statistical Manual of Mental Disorders (DSM) fourth or fifth edition (9, 15–17), Confusion Assessment Method (CAM) (10, 16), Memorial Delirium Assessment Scale (MDAS) (15, 18, 20), Delirium Observation Screening (DOS) (17), Agitation Distress Scale (20), 4 'A's Test (4AT) (21) and a chart review instrument (19). The prevalence of delirium ranged between 6.9 to 55.9%.

### Quality Assessment

The overall quality scores of the NOS scale for the included studies ranged from 5 to 8 (Table 1). Only a cohort study and a case-control study were graded as high quality (9, 10), the remaining seven studies as moderate quality (15–21). Only one cohort study achieved the maximum of four scores in the 'selection' criteria (10). The other four cohort studies failed to demonstrate that the outcome of interest was not present at the start of studies (15–18). All the three case-control studies and a cross-sectional study did not select community as controls (9, 19–21), and two of these studies did not report independent validation of delirium (19, 21). Only two studies scored maximum points in the 'comparability' criteria (9, 10). No study met all three quality criteria in the 'outcome/exposure' criteria due to lack of independent blind assessment of delirium in cohort studies and no respondents described in case-control studies. The detailed methodological quality of included studies according to the NOS scale was presented in the supplementary materials (Supplementary Table 2).

### Risk Factors

**Figure 2:**
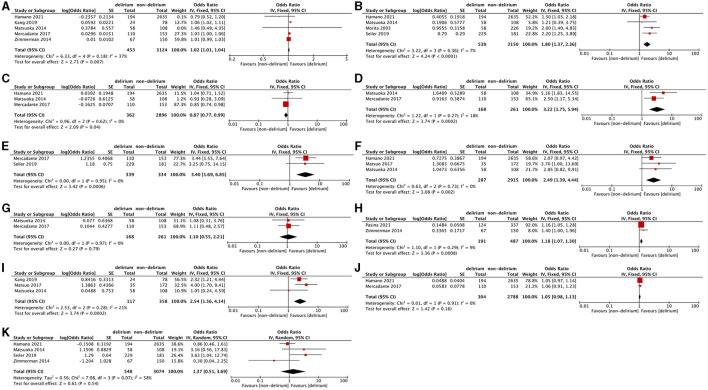
Forest plots of meta-analysis of risk factors for delirium in the palliative care population. **(A)** Age. **(B)** Male sex. **(C)** Hypoxia. **(D)** Dehydration. **(E)** Cachexia. **(F)** Opioid use. **(G)** Steroid use. **(H)** Anticholinergic burden. **(I)** Eastern Cooperative Oncology Group Performance Status. **(J)** Comorbidity. **(K)** Tumor of central nervous system.

All nine included studies explored 41 risk factors in multivariable analysis. Of these, 18 were reported as independent risk factors (Table 2). Meta-analysis was performed for 11 risk factors described in two or more studies (Figure 2).

**Table 2:** Multivariable analyses of risk factors for delirium in palliative care population.

**Risk factors**	**aOR(95% CI)**	**Reference**
Age	1.061 (1.016–1.108)	Kang et al. (16)
	1.03 (1–1.07)	Mercadante et al. (18)
Gender(male)	1.5 (1.03–2.17)	Hamano et al. (15)
	2.19 (1.251–3.841)	Seiler et al. (17)
	2.6 (1.4–5.0)	Morita et al. (20)
Brain tumor	3.63 (1.033–12.776)	Seiler et al. (17)
Hearing impairment	3.52 (1.721–7.210)	Seiler et al. (17)
Visual impairment	3.15 (1.765–5.607)	Seiler et al. (17)
Frailty	15.28 (5.885–39.665)	Seiler et al. (17)
	2.39 (1.52–3.74)	Hamano et al. (15)
Acute renal failure	16.79 (1.062–43.405)	Seiler et al. (17)
Dehydration	2.50 (1.17–5.34)	Mercadante et al. (18)
	5.16 (1.83–14.59)	Matsuoka et al. (9)
Metabolic abnormalities	4.3 (1.43–12.96)	Matsuoka et al. (9)
Hypoxia	0.85 (0.74–0.96)	Mercadante et al. (18)
Icterus	2.4 (1.3–4.4)	Morita et al. (20)
Cachexia	3.44 (1.55–7.63)	Mercadante et al. (18)
ECOG PS	2.320 (1.212–4.44)	Kang et al. (16)
	4 (1.7–9.3)	Matsuo et al. (10)
Karnofsky score	0.93 (0.87–0.98)	Mercadante (18)
Pressure sores	3.66 (1.102–12.149)	Seiler et al. (17)
Opioid use	3.7 (1.0–13)	Matsuo et al. (10)
Chemotherapeutic drugs penetrating the blood-brain barrier	18.92 (1.08–333.04)	Matsuoka et al. (9)
Anticholinergic burden	1.4 (1–1.9)	Zimmerman et al. (19)
	1.16 (1.05–1.28)	Pasina et al. (21)

*aOR, adjusted Odds Ratio; ECOG PS, Eastern Cooperative Oncology Group Performance Status*.

#### Age and Gender

Age was the most frequent risk factor discussed by five studies using multivariable analyses (9, 15, 16, 18, 19). A meta-analysis of these studies presented that older age was statistically associated with increased delirium risk (aOR: 1.02, 95%CI: 1.01–1.04, P = 0.007; *I*^2^ = 37%, *P* = 0.18). Regarding gender, the pooled analysis of four studies showed that male sex was significantly associated with delirium (aOR: 1.80, 95%CI: 1.37–2.36, *P* < 0.0001; *I*^2^ = 7%, *P* = 0.36).

#### Hypoxia

Hypoxia was discussed in three studies (9, 15, 18). One of these studies found that hypoxia is significantly associated with delirium in multivariate analysis (18).This association remained statistically significant in pooled analysis (aOR: 0.87, 95%CI: 0.77–0.99, *P* = 0.04; *I*^2^ = 0%, *P* = 0.62).

#### Dehydration

Two studies reported dehydration as an independent risk factor for delirium (9, 18). The meta-analysis of the two studies also confirmed the association between dehydration and delirium (aOR: 3.22, 95%CI: 1.75–5.94, *P* = 0.002; *I*^2^ = 18%, *P* = 0.27).

#### Cachexia

Cachexia was explored in two studies (17, 18), both of which did not mention the diagnostic criteria of cachexia. A statistical association between cachexia and delirium was found in one multivariable analysis (18) and pooled analysis (aOR: 3.40, 95% CI: 1.69–6.85, *P* = 0.0006; *I*^2^ = 0%, *P* = 0.95).

#### Medication

Three studies explored the association between opioid use and delirium (9, 10, 15).Use of opioid was significantly associated with delirium in one multivariable (10) and in pooled analysis (aOR: 2.49, 95%CI: 1.39–4.44, *P* =0.16; *I*^2^ = 0%, *P* = 0.73).The relationship between steroid use and delirium was investigated in two studies (9, 18). However, no significant association was found in multivariable analyses and the pooled analysis (aOR: 1.1, 95% CI: 0.55–2.21, *P* = 0.79; *I*^2^ = 0%, *P* = 0.97). The anticholinergic burden was discussed in two studies using the Anticholinergic Risk Scale and Anticholinergic Cognitive Burden scale, respectively (19, 21). The anticholinergic burden was identified as an independent risk factor for delirium in the two studies, which was confirmed in the pooled analysis (aOR: 1.18, 95% CI: 1.07–1.30, *P* = 0.0008; *I*^2^ = 9%, *P* = 0.29).

#### Performance Status

The performance status, a patient's ability to perform everyday activities, was routinely evaluated for survival prediction of cancer patients in palliative care settings (22, 23).Three studies (9, 10, 16) used Eastern Cooperative Oncology Group Performance Status Scale (ECOG PS) and one study (18) used Karnofsky score to rate patients' performance status. The association between performance status assessed by ECOG PS and delirium was statistically significant in two multivariable analyses and confirmed in pooled analysis (aOR: 2.54, 95%CI: 1.56–4.14, *p* = 0.0002; *I*^2^ = 21%, *P* = 0.28). Since only one study reported impaired performance status evaluated by Karnofsky score as an independent risk factor for delirium (18), we did not perform a meta-analysis.

#### Comorbidity

Comorbidity was investigated in two studies (15, 18). One study used the Charlson Comorbidity Index to evaluate comorbidity (15), while the other study did not report the tool to assess comorbidity. The association between comorbidity and delirium was not statistically significant in multivariate analysis and pooled analysis of the two studies (aOR: 1.05, 95%CI: 0.98–1.13, *P* = 0.16; *I*^2^ = 0%, *P* = 0.91).

#### Tumor of the Central Nervous System

**Figure 3:**
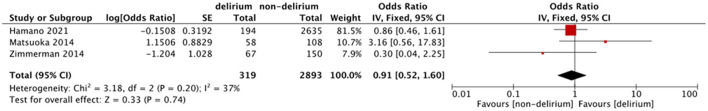
Sensitive analysis of tumor of central nervous system.

Three studies explored the association between central nervous system metastasis and delirium (9, 15, 19). Another study reported brain tumors as an independent risk factor for delirium (17). However, no significant association was found in the meta-analysis of these four studies (aOR: 1.37, 95% CI: 0.51–3.69, *P* = 0.54), while a significant heterogeneity was found (*I*^2^ = 58%, *P* = 0.07). As brain tumors included primary brain tumor and brain metastasis, we conducted a sensitivity analysis in which we excluded the study investigating brain tumors and restricted studies to central nervous system metastasis (17). Although the heterogeneity improved (*I*^2^ = 37%, *P* = 0.2), the combined odds ratio was still no significance (aOR: 0.91, 95% CI: 0.52–.6, *P* = 0.74). The result of sensitive analysis was shown in Figure 3.

#### Others

The remaining eight independent risk factors were reported only in one study: visual impairment (17), hearing impairment (17), frailty (17), acute renal failure (17), metabolic abnormalities (9), icterus (20), pressure sores (17), and chemotherapeutic drugs penetrating the blood-brain barrier (9) (Table 2). Therefore, we did not perform any pooled analyses among these risk factors.

## Discussion

Our systematic review and meta-analysis revealed that older age, male sex, dehydration, hypoxia, cachexia, using opioids, anticholinergic burden, and poor performance status were risk factors for delirium in the palliative care population, while comorbidity, tumor of the central nervous system, and use of steroid were not. The remaining eight independent risk factors including visual impairment, hearing impairment, frailty, acute renal failure, metabolic abnormalities, icterus, pressure sores and chemotherapeutic drugs penetrating the blood-brain barrier reported only in one study were not involved in pooled analyses.

Previous systematic reviews of risk factors for delirium were usually developed from studies of population recruited from surgical, intensive care and general medical settings. No systematic review has specifically evaluated risk factors for delirium in palliative care units. It is noteworthy that palliative care population with poor physical condition and multiple dimensions of distress may have unique risk factors for delirium. The European Society for Medical Oncology (ESMO) Clinical Practice Guideline (CPG) summarized direct risk factors (e.g., tumors of central nervous system, radiation to brain, chemotherapy) and indirect risk factors including physical complications, predisposing comorbidities and medications for delirium in adult cancer patients. However, the ESMO guidance did not use meta-analysis to identify key risk factors (24). A recent systematic review limited to older cancer adults receiving chemotherapy revealed six independent risk factors for delirium, but all the risk factors were not included in pooled analyses (25).Our study was the first systematic review focusing on palliative care population and use meta-analysis to evaluate risk factors for delirium, which filled a research gap on delirium.

Risk factors such as age, hypoxia, visual impairment, hearing impairment, frailty, renal failure, and metabolic abnormalities are well-recognized risk factors for delirium and demonstrated in many studies including our review (26–29). In addition, we found that certain risk factors such as dehydration and cachexia were more consistently associated with delirium in the palliative care population. Dehydration and cachexia are highly prevalent in palliative care units and associated with reduced quality of life and increased mortality (30, 31). Although the underlying mechanisms by which dehydration and cachexia cause delirium are not clear, metabolic insufficiency may be a possible explanation and could be changed by hydration and nutrition (30, 32). Hydration and nutrition have been considered effective non-pharmacologic approaches in preventing delirium in older persons (33). However, there is little sufficient or consistent evidence in palliative care population (34–36). So more clinical trials are needed to provide insight on the roles of hydration and nutrition for delirium in the future.

Symptom management is a key element of palliative care, but many drugs used to control symptoms (e.g., opioids, steroids and anticholinergic drugs) are considered to correlate with delirium (37). Opioids, as the mainstay of cancer pain management, could precipitate delirium by increase dopamine release in the nucleus accumbens through opioid receptors (38, 39), which has been confirmed in palliative care population (40–42). However, the impact of opioid dose on delirium is still controversial. Some studies considered that the risk of delirium was increased with higher doses of opioids (43, 44), but others found that receiving no opioids or very low doses of opioids could increase risk of delirium in patients with severe pain (45–47). So, pain may act as a confounding factor in the relationship between opioids and delirium. Our meta-analysis revealed that using opioids was a risk factor for delirium, but we did not evaluate the correlation between opioid dose and pain due to lack of data. As such, we should consider the possibility that the increased delirium risk may be related to refractory pain following a rapid increment in opioids consumption within a short time, irrespective of basal opioid dose. Therefore, further research is required to clarify the relationships among opioid dose, pain, and delirium. To reduce delirium occurrence, we should take some strategies, such as routine measurements for delirium, regular opioid dose titration, and adequate pain control (44). Furthermore, the opioid rotation could also be beneficial for reducing delirium since the risk of delirium may differ in various opioids due to their specific pharmacokinetic and pharmacodynamic properties (48, 49).

Anticholinergic drugs, such as scopolamine and loperamide are often used in palliative care for the anti-secretory, anti-emetic and anti-diarrheal effects. However, use of anticholinergic drugs may induce cholinergic deficiency and thus affect attention, sleep, and memory (50). The relationship between anticholinergic drugs and delirium has been investigated, but results were conflicting. This might partly attribute to different anticholinergic drug scales used in previous studies. A recent systematic review recommended Anticholinergic Risk Scale as a useful tool to identify patients at an increased risk for delirium (51). We also found that the cumulative anticholinergic burden could increase the risk of delirium. Further studies are needed to confirm it using uniform tool to measure anticholinergic drug burden, such as Anticholinergic Risk Scale. In addition, proper use of anticholinergic drugs and regular medication review may be beneficial for reducing delirium. Concerning steroids, only two studies explored the association with delirium and no correlation was found. Given the small number of included studies without mentioning drug dose and treatment course, the actual relationship between steroids and delirium in the palliative care population still needs further study.

There were still some risk factors such as male sex and poor performance status identified in our review were not exactly same as previous studies (52, 53). This could be partially explained by palliative care population we studied. More evidence is needed to draw definitive conclusions. Regarding comorbidity and tumor of the central nervous system, we did not find any significant association with delirium, which was inconsistent with previous findings (24, 26, 28). The possible reasons may be due to different appraisal of comorbidity in varied studies. Future studies should use standard measurement to evaluate comorbidity, such as Charlson Comorbidity Index. As for tumor of the central nervous system, there was substantial heterogeneity across the included studies. The different impacts of primary brain tumor and metastatic brain tumors on delirium could be responsible for this heterogeneity. Therefore, future studies are necessary to verify respective effect of primary brain tumor and metastatic brain tumor on delirium.

Despite existing uncertain risk factors, our study has important implications for clinical practice. Some risk factors identified in our meta-analysis are modifiable, for example, hypoxia, dehydration, use of opioids, and anticholinergic burden. Management of these risk factors could help prevent and improve delirium. The related strategies include oxygen therapy, hydration, nutrition, and prudent use of opioids and anticholinergic drugs. Besides this, the non-modifiable risk factors, such as age, gender, performance status are still useful for prediction of delirium in palliative care population. Overall, our findings highlight the palliative care population at high risk of delirium and suggest several directions for possible intervention.

Our study has several strengths. It was the first systematic review and meta-analysis to quantitatively summarized risk factors for delirium in the palliative care population. We performed a comprehensive literature search following rigorous selection criteria. All included studies used multivariable techniques to analyze risk factors, which ensured the reliability of the results in our meta-analysis. However, there are some potential limitations of this review. First, relatively few original studies were included by limitations in multivariable analysis. Thus, we could not perform publication bias analysis and subgroup meta-analysis. Second, few studies explicitly stated the variables that were adjusted for in multivariable analysis. Therefore, the combined results should be interpreted with caution. Third, the independent risk factors reported only in one study could not be definitively confirmed without more information.

## Conclusion

We found that older age, male sex, dehydration, hypoxia, cachexia, using opioids, anticholinergic burden, and poor performance status were risk factors for delirium in the palliative care population. These risk factors should be considered when developing strategies to prevent delirium in palliative care settings. The interaction among diverse risk factors for delirium need to be explored further.

## Data Availability Statement

The original contributions presented in the study are included in the article/Supplementary Material, further inquiries can be directed to the corresponding author/s.

## Author Contributions

DG and JY contributed to conception and design of the study. DG and TL were responsible for the study selection and quality assessment. DG and YZ contributed to data extraction. JY and LG provided overall supervision to the project. DG and CD drafted the manuscript. All authors contributed to manuscript revision, read, and approved the submitted version.

## Funding

This study was supported by Grants from National Clinical Research Center for Geriatrics, West China Hospital, Sichuan University (Z20191003, Z20192009); 1.3.5 project for disciplines of excellence, West China Hospital, Sichuan University (ZYJC21005); Sichuan Science and Technology Program (2021YFS0139); and West China Nursing Discipline Development Special Fund Project, Sichuan University (HXHL20014).

## Conflict of Interest

The authors declare that the research was conducted in the absence of any commercial or financial relationships that could be construed as a potential conflict of interest.

## Publisher's Note

All claims expressed in this article are solely those of the authors and do not necessarily represent those of their affiliated organizations, or those of the publisher, the editors and the reviewers. Any product that may be evaluated in this article, or claim that may be made by its manufacturer, is not guaranteed or endorsed by the publisher.
