# Supplementary material for: Risk Factors for Delirium in the Palliative Care Population: A Systematic Review and Meta-Analysis
Source: Front Psychiatry. 2021 Oct 21;12:772387. doi: 10.3389/fpsyt.2021.772387 (PMC8566675; doi:10.3389/fpsyt.2021.772387)
Supplement: Supplementary file 1 [file Table_1.DOCX]

Supplementary Material

# Supplementary Table1. Embase, Medline, Pubmed, and Cochrane library via OVID

| 1. exp Terminal Care/  2. exp Palliative Care/  3. exp Terminally Ill/  4. exp Hospices/  5. palliat$.mp.  6. (end adj3 life).mp.  7. (terminal* adj6 (care or caring or ill*)).mp.  8. (terminal-stage* or terminal stage* or dying or (close adj6 death)).mp.  9. hospice*.mp.  10. ((end-stage* or end stage*) adj6 (disease* or ill* or care or caring)).mp.  11. ((incurable or advanced) adj6 (ill* or disease*)).mp.  12. (dying adj3 care).mp.  13. dying.mp.  14. late stage.mp.  15. advanced directive.mp.  16. 1 or 2 or 3 or 4 or 5 or 6 or 7 or 8 or 9 or 10 or 11 or 12 or 13 or 14 or 15  17. exp Delirium/  18. deliri$.mp.  19. acute confusion.mp.  20. acute brain failure.mp.  21. acute organic psychosyndrome.mp.  22. organic mental disorders.mp.  23. acute brain syndrome.mp.  24. metabolic encephalopathy.mp.  25. ICU psychosis.mp.  26. acute psycho-organic syndrome.mp.  27. clouded state.mp.  28. clouding of consciousness.mp.  29. exogenous psychosis.mp.  30. toxic psychosis.mp.  31. toxic confusion.mp.  32. exp Alcohol-Withdrawal-Delirium/  33. exp delirium tremens/  34. or/17-31  35. 32 or 33  36. 34 not 35  37. 16 and 36 |
| --- |

# Supplementary Table 2. Methodological Quality of included studies according to the Newcastle Ottawa Scale

| Reference | Selection | Comparability | Outcome/Exposure | Total Score |
| --- | --- | --- | --- | --- |
| Hamano et al. | 3 | 1 | 2 | 6 |
| Seiler et al. | 3 | 1 | 2 | 6 |
| Kang et al. | 3 | 1 | 2 | 6 |
| Mercadante et al. | 3 | 1 | 2 | 6 |
| Matsuo et al. | 4 | 2 | 2 | 8 |
| Zimmerman et al. | 2 | 1 | 2 | 5 |
| Matsuoka et al. | 3 | 2 | 2 | 7 |
| Morita et al. | 3 | 1 | 2 | 6 |
| Pasina et al. | 2 | 1 | 2 | 5 |
